# Perceptions and Experiences of Health and Social Care Utilisation of the UK-Nepali Population

**DOI:** 10.1007/s10903-020-00976-w

**Published:** 2020-01-20

**Authors:** Bibha Simkhada, Rajeeb Kumar Sah, Alan Mercel-Sanca, Edwin van Teijlingen, Yagya Murti Bhurtyal, Pramod Regmi

**Affiliations:** 1 Department of Nursing Science, Bournemouth University, Poole, UK; 2 School of Public Health, Midwifery and Social Work, Canterbury Christ Church University, Canterbury, UK; 3 UK Nepal Friendship Society, London, UK; 4 CMMPH, Faculty of Health and Social Sciences, Bournemouth University, Poole, UK; 5 ScHARR, University of Sheffield, Sheffield, UK

**Keywords:** Black, Asian and Minority Ethnic (BAME), Health and wellbeing, Health and social care, Nepal, Inequalities in health, Mental health

## Abstract

With the growing UK Nepali community, understanding their health and social care needs is an essential to reduce health and social care inequalities. However, very little is known about the health, wellbeing and utilisation of health and social care services among the Nepali population in the UK. Therefore, this study set out to identify health and social care needs of Nepali community. The mixed-methods study was conducted with the Nepali population living in London. It consists of a semi-structured survey (N = 345); three focus group discussions and three key informant interviews. The mean age of the participants was 40.6 (± 17.6). About 28% of our sample reported having chronic health problems. About 60% currently consume alcohol and 21% were smokers. Male participants (35%) more likely to be physically active than females (21%). Registration with a family doctor/general practitioner (GP) was high (96%). However, uptake of disease screening was very low (28%). In the preceding year, 17% had experienced poor mental or emotional health. The findings also suggest language is a key barrier to utilise health and social care among UK Nepali. We suggest removing the language barrier is essential step to improve access to available health and social care services. A culturally sensitive educational initiative creating awareness about the structure of UK health and social care services is required to offer to this community.

## Background

The Nepali population in the UK predominantly comprises of the retired British Gurkha soldiers and their families, accounting for about 60% of the total Nepali population in the UK [1]. In 2008, the British Government granted Nepali British Gurkhas the right to settle in the UK [2]. The Office for National Statistics reported about 65,000 people of Nepali origin living in the England and Wales [3]. The Centre for Nepal Studies UK [4] estimated the total number to be 80,000 in 2012, whereas Nepali community organisations, including the UK Nepal Friendship Society (UKNFS), estimate the real number to be much higher in 2019.

There have been limited studies understanding the health and wellbeing of the Nepali population and little is known about their utilisation of the health and social care services in the UK [5–7]. Health services are generally freely available to all population through National Health Services (NHS). Social care services are partly freely available and partly means tested for most of the population. Adhikary et al. [7], found that only 38% Nepali in the UK were registered with a dentist. Casey [6] reported chronic illnesses such as hypertension, diabetes and obesity among the elderly and sexual health issues, alcohol/drugs, high prevalence of TB (tuberculosis) among the younger population as key problems among the UK Nepali population. There are challenges to the Nepali population [8–11] in accessing health and social care in the UK, for example as indicated in recent studies on sexual, mental health and wellbeing issues.

Moreover, the Nepali community’s lack of understanding of the NHS restricts their access to primary care, hospitals and other NHS-health and social care services. At the same time the NHS and social care services have minimal understanding and awareness of the specific health and social care needs of the UK Nepali community within the wider Black, Asian and Minority Ethnic (BAME) community. Better knowledge and understanding of the health and wellbeing of the Nepali population and their health-seeking behaviour could help to promote the uptake of health and social care. latter is important for promoting better health and the introduction of evidence-based practice to meet the needs of the Nepali community.

Hence this study aimed to: (a) identify health and social care needs of the Nepali community; (b) understand the gaps and barriers in accessing the available sexual and mental health services; (c) assess health inequalities experienced by Nepali population towards accessing health and social care services. It also intended to provide evidence based information to the NHS to engage effectively and comprehensively with the Nepali community in promoting positive healthcare for all.

## Study Design and Methods

This study was carried out among Nepali people living in the Royal Borough of Greenwich in London. This is one of the largest Nepali communities in the UK with around 4,000 people, which is about 1.7% of the borough’s total population [12]. We applied a mixed-method concurrent approach [13, 14] to collect data from Nepali population aged 18 years and above. Our participants included young people, adults, elderly, professionals, unemployed coming from diverse Nepali sub-ethnic groups that provided wide range of NHS and social care experiences and health related concerns within the community [15, 16]. The eligibility criteria for the study included individuals who lived in the UK for more than six months and who were eligible to access relevant services. Similarly health and social care professionals who were providing services to the Nepali community were eligible for the study.

For the qualitative data, we employed key informant interviews (KIIs) and focus group discussions (FGDs) in the community [17]. Two of the authors (BS + RS) conducted the interviews (n = 3) and FGDs (n = 3). The key informants included pharmacists who provided services to the Nepali population and social/community workers from the community who provided the support for the elderly population and people who lacked English language literacy. FGDs were conducted with a mixed group of elderly population and a male-only adult group (FGD participant’s age ranged between 28 and 45 years). We also aimed to interview women, despite several attempts women could not commit due to work and family commitments. All interviews and FGDs were carried out in Nepali and then transcribed and translated into English. A thematic analysis was used for the qualitative data [18]. We applied different strategies to ensure the rigour of the qualitative research process. First, we audio recorded and transcribed the interviews and focus groups word by word, next transcripts were translated by independent bilingual researcher. The transcripts were also cross checked with audio recordings to ensure trustworthiness. In addition, some of the transcripts were analysed by a second member of the team to ensure quality of the data analysis.

Similarly, the quantitative data was collected using semi-structured survey questionnaires, which was available in both Nepali and English languages. Since there was no existing questionnaire our research team designed and tested an instrument specifically for the study. The survey questionnaires were completed by the participants with the support of enumerators, while the whole process of data collection was facilitated and monitored by the lead research team. The enumerators were bilingual (Nepali and English) and were recruited from the Nepali community in Greenwich. BS and RS provided the data collection training to the enumerators, who helped them to support participants with completing the survey questionnaires in Nepali or English. The survey included information around participants’ socio-demographic information, lifestyles, health and social care service accessibility, community service use, sexual health issues, and mental health and wellbeing issues. A total number of 345 survey questionnaires were completed. However, 338 were included in this paper due to missing information in seven. We managed and analysed the data through SPSS version 21. For the health and lifestyle variables we analysed gender differences using χ^2^ tests. In Table 2, only statistically significant p-values have been reported. Qualitative and quantitative data were triangulated around the themes of access and experiences of health and social services within the Nepali community [19].

Ethical approval was obtained from Bournemouth University and individual informed consent was obtained prior to survey participation and interviews. Every precaution was taken to maintain and ensure the confidentiality and anonymity of the participants. Confidentiality within the focus groups was strictly maintained, and transcripts and tapes were also encoded to ensure anonymity.

## Findings

The key findings from the quantitative and qualitative analysis are presented under five domains.

### Socio-Demographic Characteristics and Common Health Problems

A higher proportion of men (61%) completed the survey questionnaires compared to women (39%). Mean age of the research participants was 40.6 (± 17.6) years with female participants being younger than males (mean 38.2 ± 15.6 vs 42.2 ± 18.4). Over half (58%) of our respondents were permanent residents in the UK. Most respondents (72%) were employed with 46% working full time, 68% had an annual income of less than £10,000 per year, and 46% had A-level education; just 26% were university graduates. We asked respondents “How do you rate your English language skill?”. Only 34% reported that they were fluent in English, the majority having poor English language capacity, mainly women and the elderly. About 26% lived in their own house/flat and 68% of participants lived in rented accommodation: very few (6%) lived in council housing.

Discussions with key informants and FGD participants indicated many elderly were living in an inappropriate accommodation, especially those with poor mobility and chronic health conditions:

…It is difficult to live in a shared house…Main problem is in using toilet… There is one toilet and many people are living in one house....a friend living in the shared house, she has one room upstairs. She has problem in her leg. She find difficult to walk upstairs. She has to cook food downstairs and take food upstairs to eat in her room **(FGD with elderly, 2)**.

…majority of the elderly in this area are living in very poor houses…most are in shared houses. Landlords are not treating them very well as they are splitting one room into two rooms and charging same rent as bigger room for tiny room. Some houses I have visited do not have a single window and there was not good ventilation. They hardly have space to walk. There are about 9-10 people in one house with one toilet **(Volunteer Social Worker)**.

Table 1 shows the most common health problems among participants, 28% reported chronic health problems, including high-blood pressure, diabetes, high cholesterol, asthma and TB.

**Table 1:** Top 5 common health problems (self-reported)

Health problem	Yes N (%)	Male N (%)	Female N (%)
High blood pressure	62 (18.3)	38 (18.3)	24 (18.5)
Diabetes	43 (12.7)	28 (13.5)	15 (11.5)
High cholesterol	23 (6.8)	12 (5.8)	11 (8.5)
Asthma	14 (4.1)	8 (3.8)	6 (4.6)
Tuberculosis	5 (1.5)	3 (1.4)	2 (1.5)

### Lifestyle Related Behaviour

Table 2 shows lifestyle-related behaviour, incorporating information and discussions about dietary habits, physical activity, smoking, alcohol consumption and drugs. Please note all behaviours are self reported. Just over one third (35%) of male participants had more than 6 h of physical activity compared to only 21% of women. A large number of the Nepali population rely on walking as their main form of exercise. Vigorous exercise such as attending a gym, swimming, running or playing sports, were more common in males compared to females. Our study revealed only 50.3% reported they consumed five or more portions of fruit and vegetables daily.

**Table 2:** Lifestyle related behaviour

Variables	Yes (%)	Male (N = 208)	Female (N = 130)	*p*-value
Physical activity per week				
Never	65 (19.2)	31 (14.9%)	34 (26.2%)	0.015
1–5 h	173 (51.2)	104 (50.0%)	69 (53.1%)	NS
6 or more hours	100 (29.6)	73 (35.1%)	27 (20.8%)	< 0.005
Type of physical activity				
Walking	209 (61.8)	128 (61.5%)	81 (62.3%)	NS
Gym	78 (23.1)	62 (29.8%)	16 (12.3%)	< 0.001
Swimming	45 (13.3)	32 (15.4%)	13 (10.0%)	NS
Running	72 (21.3)	57 (27.4%)	15 (11.5%)	< 0.001
Play sport	48 (14.2)	45 (21.6%)	3 (2.3%)	< 0.0001
Exercise at home	73 (21.6)	41 (19.7%)	32 (24.6%)	NS
Yoga	6 (1.8)	3 (1.4%)	3 (2.3%)	NS
Dietary habit (per day)				
Intake of more than five portions of fruit and veg	170 (50.3)	113 (54.3%)	57 (43.8%)	0.07
Smoking	70 (20.7)	59 (28.4%)	11 (8.5%)	< 0.0001
Alcohol consumption	204 (60.4)	144 (69.2%)	60 (46.2%)	< 0.0001

***P < 0.0001, **P< 0.001, *P < 0.005

*NS* non-significant

Over 60% of participants were currently alcohol users, 21% were smokers and about 10% were smokeless tobacco users that included chewing tobacco (*Surti/Khainee*), Areca nut (*Supari*), Betel Leafs (*Pan*), Gutkha (a powdery, granular white substance). About 28% of men were currently smoking cigarettes compared to 8% of women, and 70% of males consumed alcohol compared to 46% of females. About 30% in our study reported the level of alcohol intake as harmful to their health. Few participants reported using illegal drugs. Although one local pharmacist expressed concerns about widespread drug misuse among the younger Nepali population, less than 2% reported they use illegal drugs.

### Health Services Use: Satisfaction and Barriers

The qualitative data revealed significant barriers to uptake of available support services (rehabilitation, etc.) highlighting: lack of knowledge (how and why specific drugs were harmful), fear of drug use disclosure, censure from family, and feelings of humiliation and guilt were the main concerns, as evidenced below:

…a lot of people (Nepali) do not know about the service. They only come in when they are really struggling. There are only 10-11 people in therapy programme...Nepali people feel more shameful to seek support when they have problem. They do not use services, as much as other drug addict, as they have to hide it from their parents and friends… **(Pharmacist 1)**.

Although over 96% of participants reported to have registered with an NHS General Practitioner (GP) also refer to family doctor. Only 39% had 1–2 GP visits in the last 12 months: 28% did not visit their GP at all, and 6% had a very high GP attendance with 11 or more consultations. Only 45% were registered with dentists. About 38% of respondents had wellbeing check-ups, such as screenings, blood sugar monitoring and cholesterol measurement. The uptake of disease screening was very low (28%); only 25% of female participants had had cervical smear tests, and only 10% had undergone breast screening: 35% of respondents had travel vaccines before travelling to Nepal. Only a third of participants made their first choice seeing an NHS doctor/GP when medical problems manifested,. Self-medication and asking friends or families for medical advice were more popular choices (Table 3), for example: Ayurvedic medicine is popular as revealed in FGDs among both younger and older adults.

…I always give first preference to the Ayurvedic medicine .. but if it’s like I can’t stand the problem then of course allopathic medicine is the fast healer and I go for it .. Ayurvedic is a slow healer. But if there is time and can resist the problem then I prefer to use Ayurvedic medicine **(FGD with young male)**.

…we brought it (Ayurvedic medicines) when we came, it is for arthritis. It is common practice to use Ayurvedic medicine for arthritis. GP do not give any medicine for minor illness and even pharmacy do not give any medicine without GP's signature **(FGD with mix elderly 1)**.

**Table 3:** Priority for medical advice when unwell

Priority list when unwell	1st choice (N = 336)	2nd choice (N = 290)	3rd choice (N = 228)
See a physician/doctor in UK	110 (33%)	64 (22%)	70 (31%)
Self-medication/using western medicine	85 (25%)	45 (16%)	31 (14%)
Ask friend or family in UK for medical advice	73 (22%)	49 (17%)	39 (17%)
See a pharmacist in UK	38 (11%)	103 (36%)	42 (18%)
Use ayurvedic, herbal/alternative therapy	18 (5%)	12 (4%)	23 (10%)
Ask friend/family in Nepal for medical advice	6 (2%)	13 (4%)	16 (7%)
Call a doctor/clinician back in Nepal	6 (2%)	4 (1%)	7 (3%)

Almost half of the participants (49%) were satisfied with the health services, a third (37%) had no opinion while only a minority were not satisfied with the health services. The participants reported the various reasons for dissatisfaction (Table 4).

**Table 4:** Reasons for dissatisfaction with service provided

Reasons for dissatisfaction	Frequency (N = 75)
Doctor did not explain my condition and treatment well	18 (24%)
No arrangement for further treatment provided	17 (23%)
The Doctor didn’t listen to my problems well	12 (16%)
Language barrier (diagnosis/advised treatment comprehension and explanation confusing/unclear [hence difficult to reliably/safely follow]	10 (13%)
Nurses were unfriendly, rude and lack of respect	10 (13%)
Appointment delayed/appointment system [confusing]	4 (5%)
Long waiting time	4 (5%)

Interestingly for the community and the NHS, four of the top five reasons (Table 4) for dissatisfaction were related to communication. The qualitative information showed that participants had mixed experiences from accessing health services within Southeast London, the study area. Participants valued the care and treatment they received, however many expressed language barriers as a key concern for accessing health and social care services. Elderly participants, in particular, reported their negative experiences and highlighted the key issues were delayed appointments, concerns related to issuing prescriptions (clarity on dosage, side-effects, etc.) costs and language barriers.

…the main problem we are facing is to access the doctors. They just give us appointment after one week…In emergency we cannot wait that long. We are unwell now, **by the time we get appointment we might be dead**…... If we run out medicine we have to see doctors. Only doctors can give medicine otherwise we will be without medicine. We need to buy medicine costing 7-8 pounds which is bit expensive. **We need someone who can speak Nepali and explain our problems for us** **(FGD with mixed elderly 1)**.

…I did not get interpreter when I had to see doctor and I needed interpreter. I could not explain very well. I knew few words and that helped me in emergency and got help. … They said to me that I have to stay in the hospital for two weeks. My wife cannot do anything; she cannot prepare dinner for her. I told them that I cannot leave my wife without anybody. I could not explain more and started to use body language still they did not understand. Luckily I met one Nepali cleaner and request him to explain my situation. He explained everything to nurse. We are struggling everyday due to language. It is very difficult. I even could not tell I cook dinner for my family **(FGD with mix elderly 1)**.

The discussions with the pharmacist revealed similar concerns; however, they highlighted that poor knowledge and understanding of the NHS in general (how it works, what it comprises of, services access, complaining, engaging, etc.): in particular about availability and accessibility of health and social care services to the elderly population were the key reasons behind their dissatisfaction.

I think they actually do not understand the NHS, it is quite difficult to engage with them, they might feel we are difficult and not supporting them when they run out their medicine, they demand medicine immediately in the pharmacy but we have to follow protocol and we have to wait for doctor’s prescription. Some of them are recently migrated and when they feel cold they ask for antibiotics. We cannot just give them; there is problem of antibiotic resistance. We need to be very careful and we cannot give antibiotic for viral infection. I think they are used to that kind of service. They asked for stronger medicine and they even said that Paracetamol won't work. It is very hard to explain them. I also found that they did not know to switch pharmacy where they wish to go where they are more comfortable and close for them. They think it is big issues and said that they have to ask other pharmacist and family members. Even I said that you have right to change your pharmacist as per your conveniences and you can get you medicine anywhere. I do not think they can access all the available services that are important for them **(Pharmacist 2)**.

This study also highlighted the cultural issues around accessing healthcare services in Nepal, which together with the language barrier can shape the expectations of the elderly population.

…Due to difference in the cultural background that plays another role.. In Nepali context we always look for prompt recovery... here it is more like slow recovery and that may be the difference too .. the system is different here and so sometimes we do not wish to wait for longer and we feel more difficult about it which reminds us to think that it was better at home as you could get the service immediately whenever you want, if you pay money. With money you get any treatment at any time but here due to long queues there are waiting times” **(FGD with young male)**.

### Sexual and Reproductive Health (SRH): A Cultural Taboo

Most participants felt uncomfortable with the sexual and reproductive health section of the questionnaire. Only 6% reported to have sexual health problems and only 2% of participants (all males) reported having more than one sexual partner. The most common family planning method was the condom (77% of those who used any family planning method). Implant and the pill were also used, and only 1% was sterilised. All participants reported themselves as heterosexual: as academic research and diversity monitoring in the UK [Stonewall, etc.] and globally, indicates always at least 6% of the total population are LGBT, this finding indicates fears for social and cultural reasons, even in the UK of UK Nepali LGBTs self-disclosing.

The qualitative interviews revealed that cultural issues, concerns about privacy-confidentiality, were barriers restricting discussion about and use of sexual and reproductive health services. Interviewing a pharmacist providing services to SE London Nepali community members highlighted cultural taboos and fear of disclosing confidential information about sexual health were the main reasons for low uptake:

…they are not using the sexual health services… Maybe due to cultural reasons and I think obviously when they see another Nepali that more at sometimes frighten them up… I know that Nepali community are quite close... when you are younger.. they have a different mentality.. they understand that they need to get something…they need to speak to someone then they will whereas I think elderly are a little bit more shy. Should you want to call it or uncomfortable actually.. aahh.. to speak to a Nepali personally about it… so it’s a bit aged I think they can be a little bit uncomfortable **(Pharmacist 2)**.

An interview with a pharmacist also highlighted that lack of knowledge and ignorance were the main reasons for poor use of contraception.

…Mainly Nepali come and ask for abortion request and ask morning after pill. They come for treatment rather than prevention. They come too late; it would be good if they use contraception in the beginning. Some of them come and asked for abortion places. They come very late and they do not know sexual health clinic. ..I do not think they have very good knowledge on these services **(Pharmacist 1)**.

### Mental Health and Wellbeing

About 17% respondents reported to have experienced poor mental or emotional health in the last 12 months, particularly the elderly. Only 8% of total participants reported they were aware of local mental health support and services; however, the uptake of mental health services was very low. The FGDs revealed that economic hardship, family/relationship problems, language barriers, cultural differences, feelings of loneliness and extreme weather in the UK were major reasons behind their poor mental wellbeing.

…elderly people are finding difficult to interact with others. Most of the elderly cannot speak English, they do not have families. They are very lonely. Another things, there is no living room to sit and talk to others and they do not have TV in the house too. There is nothing for them to entertain or spend their time **(Volunteer Social Worker)**.

The interviews with pharmacists highlighted under-reporting of mental health issues due particularly to cultural taboos affecting the support they receive.

…we have not got people coming to us complaining mental health issues… I think it is bit difficult for them to admit their weakness. It may be due to their cultural things. … only mentioned about it when it is extreme case. It is only known when they attempted suicide and postpartum psychosis. It may be useful if they could use the services in early stage and they could not have reach to attempt suicide…they use services only when they are in crises and very last minute **(Pharmacist 1)**.

…I think that mental health is a big issue... again it’s that taboo... isn’t it? They don’t want to say there is something wrong or if they are depressed or ... they just don’t want to admit it … is that strong heart? I think it’s important that mental health because .... you don’t want to go under depression and stuff like that .. it can be prevented **(Pharmacist 1)**.

## Discussions

To our knowledge, this study is first of its kind to explore health and social care needs of Nepali communities in the UK. Understanding health and social care needs of migrant populations (especially mainly first generation) is an important to reduce health and social care inequalities [20, 21]. There are various social, cultural and lifestyle factors that determine health and wellbeing of the population [22]. This study demonstrated that the majority of the Nepali population, although in employment and well educated, have low income. Low income lead to adoption of and entrapment in poor lifestyles that eventually have negative impacts on health and wellbeing [23] and worsened when compounded by the language barrier, especially for women and the elderly population. Poor English language skills act as major real-life barriers towards accessibility of health and social care services, including appropriate shelter.

Our study revealed the majority of Nepali older people lived in poor quality rented and shared accommodation due to lack of negotiating [language skill] ability to select appropriate accommodation or access council housing. Quality of housing is a fundamental for good physical health and mental wellbeing and essential for reducing health inequalities [24]. Stress relating to having poor housing or rented accommodation and lack of housing arrangements from local authorities, possess significant health-harming risks for women, the elderly and those with long-term health conditions [25]. Our study suggests that at the very least the housing section of the local council should assess whether or not housing is suitable for the elderly population or those with long-term health conditions. Likewise, the health and social care needs of the Nepali community need to be practically understood through engaging directly with local communities and NHS & local authority health and social care service providers. This must be seen from wider perspectives such as age, gender, sexual orientation (women & LGBTs face particular additional issues and challenges), degree of cultural acclimatisation and ease in understanding and communicating in English language.

As highlighted above, the lower socio-economic situation contributes towards poor lifestyles (as does experience of discrimination and marginalisation), as evident in this research, seen for example through the majority of the Nepali population having limited physical activity, with ‘walking’ being the preferred exercise choice. This study is consistent with studies conducted on other South Asian ethnic groups in England [26], Nepali males are more likely to go for gym and running in compared to female counterpart. It is interesting to note Nepali adults eat a more healthy diet in comparison to the UK average population. Our study found that over 50% of Nepali adults eat five or more portions of fruit and vegetable whereas only 26% of all adults eat five portions of fruit and vegetables per day in the UK [27]. Our study found a relatively higher proportion of UK Nepali consumed alcohol, smoked tobacco and raised concerns about the issues related to drug abuse, particularly among young people. Although two thirds of participants were currently drinkers, only a third recognised their alcohol intake level was harmful to their health. Males consumed higher amounts of alcohol compared to female counterparts, which is consistent with other communities [28]; however, Nepali men in this study drink much higher (about 71%) than men from other communities and nationally (about 57%) in the UK (3). Alcohol use amongst the Nepali population is becoming very common [29]. Parajuli et al. [30]) argue that globalisation, Western consumer culture and economic liberalization have resulted in normalisation of alcohol usage within the wider Nepali society. The cultural acceptance of alcohol consumption within the Nepali community has created a blurred line between acceptable levels of drinking and excessive alcohol consumption [31]. The main concerns are that the UK Nepali population members fail to correctly assess the health risk implications of their drinking habits.

Literature shows that minority ethnic groups in the UK are under-represented in seeking treatment and advice for drinking problems [32]. Therefore, there is an urgent need for alcohol consumption risks awareness particularly among Nepali men, as alcohol-related death and disability is rising worldwide which is the eighth leading cause for death and fifth for disability [33]. We found the use of illegal drugs to be very low among our participants, although key health workers suggested it may be higher in the Nepali community than in the general community. This low number could be because of willingness to hide this habit from family, friends and society. The issues of alcohol consumption, smoking and drug abuse were in line with other deprived populations in UK, which meant the Nepali community requires comparable support to create awareness about these issues to safeguard young people from risky health-harming behaviour and the older population from poor health outcomes [20].

Chronic health conditions, such as cardiovascular disease, diabetes and asthma are most common health problems among the Nepali population in the UK, which is associated with lower socioeconomic status and poor lifestyle choices [34]. It is essential for those suffering from chronic health conditions to be aware of availability and accessibility of health and social care services. The majority of Nepali participants were registered with a GP, however, very few accessed these. Particularly, older people seem to access health and social care services rarely due to various barriers, some of which included the waiting time to see their GP, lack of knowledge about healthcare services, and language barriers [35]. The findings suggest that the Nepali elderly population expected immediate access to NHS health services, which when found wonting, may have prompted them to self-medicate, ask friends or families for medicine or use *Ayurveda* medicine during sickness rather than waiting to see the GP. This shows poor knowledge and understanding about NHS services among the community in the UK, which is also demonstrated through the low uptake of screening services and travel vaccines [36, 37]. Others who used health services expressed dissatisfaction about [NHS to community members] poor communication, often linked with language barriers and not uncommonly unfriendly behaviour from NHS administrative staff. This clearly indicates that the NHS Equality Delivery System (EDS) lacks effective implementation. Similarly, we found poor use of dental services among the Nepali community, as reported previously [7]. The barriers to NHS dental health services were mostly associated with lack of need and affordability of the dental care.

Our findings confirm the Nepali community is unambiguously reluctant in sharing information about sexual and mental health wellbeing; clearly mirrored during interviews and FGDs with the participants. However, this is unsurprising as sex and sexuality are still considered taboo subjects in Nepali society [11]. Sexual and mental health problems are complex and people from the South Asian community fear to disclose personal and confidential information to anyone whom they do not know [10, 11, 38]. This suggests the Nepali population may not seek sexual and mental health services within the community due to stigma and cultural taboos associated with revealing any sexual and reproductive health or mental health issues [8, 39].

However, the key health informants and the volunteer social workers highlighted that sexual, reproductive health and mental health issues were hidden within the Nepali community. Some participants who responded to the questionnaires and discussed about mental health issues associated the experience of poor mental health with social isolation and feeling of loneliness due to language barriers and cultural differences, which is in line with other studies [40, 41]. Actual or perceived mental health problems have negative impacts on how those community members associated or are appearing to be associated with such problems, are seen in the community with concomitant negative impacts on their status in the South Asian community [42], which may have discouraged Nepali people from utilising available mental and sexual health services. Jolly [43] highlighted that UK healthcare providers had limited understanding about supporting mental health issues among Nepalese population (clearly this wont of knowledge will extend to the broader South Asian Community). Cultural integration and the issues of privacy and confidentiality, therefore, need to be considered while delivering services on these sensitive issues.

Our research indicates need for a community-wide educational initiative intervention creating awareness about the structure of health and social care services, on the uses of those services, with a lead by and support from national organisations like Public Health England and NHS England in partnership with the Nepali community, required to deliver culturally-sensitive health and social care services to community members in the UK, helping thereby to promote awareness about their physical and mental health issues and well-being. It should further include appropriate support for UK Nepali lesbians, gays, bi-sexual and transgender people (LGBT) who are a needful, yet as our research showed, invisible population in the community, with particular vulnerabilities, and needs relating to sexual and mental health.

## Conclusion

With the growing Nepali community in the UK, understanding community members’ health and social care needs is essential to reduce health and social care inequalities in the UK. There are many socio-cultural and language issues that impact at practical level health/medical and social care provision levels in the Nepali community, particularly among its elderly population, impeding health and social care service uptake by Nepali community. In the area of mental health and sexual health there are cultural taboos which makes it difficult for Nepali migrants to disclose for example issue about sex or sexuality or talk about mental health problems. In order to tackle health inequalities to improve health and wellbeing of Nepali population, overcoming the language barrier is a first step to improve access to available health and social care services. Importantly, lifestyle related issues such as high consumption of alcohol and tobacco consumption is another issue that should be studied in detail to understand the impact of alcohol and tobacco consumption on individual health and impact on society.
